# Resonant third-harmonic generation driven by out-of-equilibrium electron dynamics in sodium-based near-zero index thin films

**DOI:** 10.1515/nanoph-2023-0743

**Published:** 2024-01-15

**Authors:** Matteo Silvestri, Ambaresh Sahoo, Luca Assogna, Paola Benassi, Carino Ferrante, Alessandro Ciattoni, Andrea Marini

**Affiliations:** Department of Physical and Chemical Sciences, University of L’Aquila, Via Vetoio, 67100 L’Aquila, Italy; CNR-SPIN, c/o Dipartimento to di Scienze Fisiche e Chimiche, Via Vetoio, Coppito, L’Aquila 67100, Italy

**Keywords:** third-harmonic generation, epsilon-near-zero, ultraviolet, plasmonics, nonlinear optics, out-of-equilibrium

## Abstract

We investigate resonant third-harmonic generation in near-zero index thin films driven out-of-equilibrium by intense optical excitation. Adopting the Landau weak coupling formalism to incorporate electron–electron and electron–phonon scattering processes, we derive a novel set of hydrodynamic equations accounting for collision-driven nonlinear dynamics in sodium. By perturbatively solving hydrodynamic equations, we model third-harmonic generation by a thin sodium film, finding that such a nonlinear process is resonant at the near-zero index resonance of the third-harmonic signal. Thanks to the reduced absorption of sodium, we observe that third-harmonic resonance can be tuned by the impinging pump radiation angle, efficiently modulating the third-harmonic generation process. Furthermore, owing to the metallic sodium response at the pump optical wavelength, we find that the third-harmonic conversion efficiency is maximised at a peculiar thin film thickness where evanescent back-reflection provides increased field intensity within the thin film. Our results are relevant for the development of future ultraviolet light sources, with potential impact for innovative integrated spectroscopy schemes.

## 1 Introduction

Nonlinear (NL) radiation-matter interactions give rise to a wide range of diverse physical phenomena, e.g., frequency conversion [1], all-optical self-effects [2], generation of non-classical radiation [3], and many others. In particular, harmonic generation in photonic materials provides fundamental insights into quantum mechanical processes, and further offers a promising platform to devise compact ultraviolet (UV) radiation sources [4], [5]. Artificial photonic materials with low dielectric permittivity, known as epsilon-near-zero (ENZ) metamaterials [6]–[8], enhance the inherently weak NL interaction producing extreme NL dynamics [9], second and third-harmonic generation (THG) [10], and provide active control of tunneling [11], and optical switching [12]. Furthermore, they naturally bypass phase-matching requirements owing to the large effective wavelength ensuing at ENZ conditions, thus leading to high THG efficiency [13]. Moreover, ENZ materials can also naturally exist in the form of plasmas, transparent conductors, and metals near their bulk plasma frequency [14]. ENZ media are currently adopted for vortex generation applications [15] and for sensing, guiding, trapping, and emission of visible/infrared (IR) radiation [16]. The ENZ extraordinary boosting of third-order nonlinearity in ENZ thin films [17] ensues from the combined effect of (i) field enhancement for transverse magnetic (TM) excitation, (ii) increased averaged field intensity due to amplified effective wavelength, and (iii) slowdown of light propagation enabling nonlinearity accumulation over time [18]–[22]. Furthermore, amplified Kerr nonlinearity in ENZ conditions enables light-induced “metal–dielectric” transition [23], [24] producing self-organization of frozen light into still solitary spatial doughnuts [25]. Currently adopted ENZ media for enhanced NL optics mainly focus in the near-IR part of the spectrum, where doped semiconductors, oxides, and nitrides display ENZ features [14]. Moreover, poor metals exhibit marked ENZ response in the UV [14] and are promising for manipulation and guidance of UV radiation [26]. Sodium (Na) is a particularly promising UV-ENZ material displaying “metal–dielectric” crossover wavelength at *λ*_ENZ_ ≃ 230 nm and an imaginary susceptibility of two orders of magnitude smaller than typical IR-ENZ media like indium–tin–oxide (ITO) [27], thus undergoing mitigated absorption. As a drawback, because Na interacts strongly with air and water, its practical implementation in NL optics applications is challenging. Recently, however, thermo-assisted spin-coating [28] and phase-shift photo-lithography [29] have been adopted to fabricate high-quality Na films, enabling surface plasmon polaritons [30] and thermosensitive plasmonic color [31].

Here, we investigate the potential of Na-based thin films for THG applications at the nanoscale. We model NL electron dynamics in Na by the Fokker–Planck–Landau (FPL) theoretical framework [32], a well-established classical approach in out-of-equilibrium (OOE) statistical mechanics to model plasmas. We calculate analytically the Landau collisional integral in the weak coupling limit [33], obtaining a set of hydrodynamical equations (HDEs) accounting for NL electron dynamics in Na. We emphasize that our HDEs account for damping and heating saturation occurring when OOE instantaneous electron velocities become higher than the thermal velocity owing to collision quenching [33]. By solving HDEs perturbatively, we derive an analytical expression for the dependence over the pump field angular frequency *ω* = 2*πc*/*λ* of the collision-driven THG NL-susceptibility $\chi_{\text{coll}}^{\left(3\omega \right)}\left(\omega \right)$ , where *c* is the speed of light in vacuum and *λ* is the pump wavelength. Finally, by solving the scattering problem of the pump field in the undepleted pump approximation, we calculate the THG NL-polarization within the Na-based thin film and model its radiation of *λ*_THG_ = *λ*/3 forward and backward waves, accounting for multiple reflections of the THG signal. We observe that, when the THG wavelength *λ*_THG_ matches the angle-dependent near-zero index (NZI) dispersion curve of the Na-based thin film *θ*_NZI_(*λ*_THG_), the THG process becomes resonant for TM pump excitation due to the enhanced radiation amplitude of microscopic dipoles oscillating at *λ*/3 in an ENZ environment. Moreover, for both TM and transverse-electric (TE) pump excitation, we observe THG enhancement for *λ*_THG_ < *λ*_ENZ_ due to Fabry–Perot resonances of the Na-based thin film, which becomes transparent in such a spectral range. We illustrate the dependence of the THG process over the Na-based thin film thickness *d* and over the pump impinging angle, wavelength and intensity. Such a systematic analysis unveils that THG is maximised at a peculiar thin film thickness $\bar{d}≃40$ nm where evanescent back-reflection enhances the average field intensity in the thin film.

## 2 OOE electron dynamics in Na

We model OOE electron dynamics in Na by classical kinetic theory, where the system is regarded as a dilute electron gas with number density *n*(**r**, *t*) immersed in an idealized homogeneous background of positive uniform density *n*_0_, physically accounting for lattice ions of mass *M* staying at equilibrium temperature *T*_0_. The time-dependent electron distribution function *f*(**r**, **w**, *t*) is defined from the infinitesimal probability d^6^ *P* = (1/*N*)*f*(**r**, **w**, *t*)d^3^ *r*d^3^ *w* that an electron in Na with effective mass *m* ≃ 1.184 × 10^−30^ [34] at time *t* is placed within a volume element d^3^ *r* around **r** and has a velocity within a velocity-space element d^3^ *w* around **w**, where *N* is the total electron number within the system volume *V*. Owing to the large inertia of the lattice ions, we neglect their motion upon external electromagnetic (EM) excitation, so that their distribution function $f_{0}\left(r,w\right)=n_{0}{\left(M/2\pi k_{b}T_{0}\right)}^{3/2}\exp\left[-Mw^{2}/2k_{b}T_{0}\right]$ stays at equilibrium, where *k*_*b*_ is the Boltzmann constant. The electron distribution normalization ∫*f*(**r**, **w**, *t*)d^3^ *r*d^3^ *w* = *N* accounts for the system charge neutrality dictated by *e*∫d^3^ *r*d^3^ *w*(*f*_0_ − *f*) = 0, where −*e* is the electron charge. Within the kinetic theory framework, the temporal evolution of *f*(**r**, **w**, *t*) is governed by the Boltzmann equation

(1) $$\partial_{t}f+w\cdot \nabla_{r}f+\frac{1}{m}F_{w}\left(r,t\right)\cdot \nabla_{w}f={\left(\partial_{t}f\right)}_{\text{coll}},$$

where **F**_**w**_(**r**, *t*) = −*e* **E**(**r**, *t*) − *e* **w** × **B**(**r**, *t*) is the external EM force exerted by the electric **E**(**r**, *t*) and magnetic **B**(**r**, *t*) pump fields, and ${\left(\partial_{t}f\right)}_{\text{coll}}={\left(\partial_{t}f\right)}_{\text{coll}}^{\text{el}-\text{el}}+{\left(\partial_{t}f\right)}_{\text{coll}}^{\text{el}-\text{ph}}$ is the total collision rate resulting from electron–electron (el–el) $\left[{\left(\partial_{t}f\right)}_{\text{coll}}^{\text{el}-\text{el}}\right]$ and electron–phonon (el–ph) $\left[{\left(\partial_{t}f\right)}_{\text{coll}}^{\text{el}-\text{ph}}\right]$ collisions. While in principle the numerical solution of the Boltzmann equation provides the system’s EM response, it is computationally demanding and lacks insights into NL dynamics. To address electron collisions, the relaxation time approximation (RTA) simplifies the collision integrals but overlooks the NL dependence of ${\left(\partial_{t}f\right)}_{\text{coll}}$ over *f*(**r**, **w**, *t*). In order to model collision-driven NL electron dynamics, we evaluate ${\left(\partial_{t}f\right)}_{\text{coll}}$ in the weak coupling assumption where only grazing-angle collisions are considered and improbable frontal collisions are disregarded [33], obtaining

(2a) $${\left(\partial_{t}f\right)}_{\text{coll}}^{\text{el}-\text{el}}=\frac{C_{\text{ee}}}{m^{2}}\nabla_{w}\cdot \left\{\left[D\cdot \nabla_{w}-2\nabla_{w}\alpha \right]f\right\},$$

(2b) $${\left(\partial_{t}f\right)}_{\text{coll}}^{\text{el}-\text{ph}}=\frac{C_{\text{ep}}}{m^{2}}\nabla_{w}\cdot \left\{\left[D_{0}\cdot \nabla_{w}-2\frac{m}{M}\nabla_{w}\alpha_{0}\right]f\right\},$$

where the *C*_ee,ep_ parameters account for el–el and el–ph collisions, $D\left(\beta \right)=\nabla_{w}\nabla_{w}\beta$ and $D_{0}=D\left(\beta_{0}\right)$ are diffusion tensors, *α*(**r**, **w**, *t*) = ∫d^3^ *w*_1_ *f*(**r**, **w**_1_, *t*)/|**w** − **w**_1_| and *β*(**r**, **w**, *t*) = ∫d^3^ *w*_1_ *f*(**r**, **w**_1_, *t*)|**w** − **w**_1_| are the Rosenbluth potentials [32], *α*_0_ = *α*(*f*_0_), and *β*_0_ = *β*(*f*_0_). In this assumption, we solve Eq. (1) by the method of moments and we truncate the ensuing hierarchy of equations at the second moment, obtaining the approximate solution

(3) $$f\left(r,w,t\right)≃\frac{n\left(r,t\right)m^{3/2}}{{\left[2\pi k_{b}T_{e}\left(r,t\right)\right]}^{3/2}}e^{-\frac{m|w-v\left(r,t\right){|}^{2}}{2k_{b}T_{e}\left(r,t\right)}},$$

where *n*(**r**, *t*) = ∫*f*(**r**, **w**, *t*)d^3^ *w*, *n*(**r**, *t*)**v**(**r**, *t*) = ∫**w** *f*(**r**, **w**, *t*)d^3^ *w* is the current density, and (3/2)*n*(**r**, *t*)*k*_*b*_ *T*_*e*_(**r**, *t*) = (*m*/2)∫|**w** − **v**|^2^ *f*(**r**, **w**, *t*)d^3^ *w* is the OOE energy density. In turn, the OOE temperature of the electron gas *T*_*e*_(**r**, *t*) is defined starting from the OOE energy density, and the moments *n*(**r**, *t*) (zero-order), **v**(**r**, *t*) (first-order), and *T*_*e*_(**r**, *t*) (second-order) satisfy the hierarchy of HDEs

(4a) $$\partial_{t}n+\nabla \cdot \left(nv\right)=0,$$

(4b) $$\partial_{t}v+\left(v\cdot \nabla \right)v+\frac{3k_{b}}{mn}\nabla \left(nT_{e}\right)=\frac{1}{m}F_{\text{eff}}-\gamma F_{\gamma }v,$$

(4c) $$\partial_{t}T_{e}+\frac{2}{3}T_{e\nabla }\cdot v+v\cdot \nabla T_{e}=Q,$$

where **F**_eff_ = −*e* **E** − *e* **v** × **B** is the external effective force, *γ* ≃ 24.6 ps^−1^ (depending only over the el–ph collision parameter *C*_ep_) is the linear depolarization rate of Na [27], $v_{T}\left(T_{e}\right)=\sqrt{2k_{b}\left(T_{0}/M+T_{e}/m\right)}$ is the electron thermal velocity, *v*_T0_ = *v*_T_(*T*_0_), *γ*_th_ = 2*mγ*/(*m* + *M*) is the hot-electron linear relaxation rate, and

(5a) $$F_{\gamma }\left(v,T_{e}\right)=\frac{3v_{\text{T0}}^{3}}{2v^{2}v_{T}}\left[G\left(v/v_{T}\right)-e^{-\frac{v^{2}}{v_{T}^{2}}}\right],$$

(5b) $$Q\left(v,T_{e}\right)=\gamma_{\text{th}}\frac{Mv_{\text{T0}}^{3}}{2k_{b}v_{T}}\left[G\left(v/v_{T}\right)-\frac{T_{e}v_{\text{T0}}^{2}}{T_{0}v_{T}^{2}}e^{-\frac{v^{2}}{v_{T}^{2}}}\right],$$

(5c) $$G\left(v,T_{e}\right)=\sqrt{\pi }v_{T}erf\left(v/v_{T}\right)/2v.$$

Note in the expressions above that $F_{\gamma }\left(v,T_{e}\right)$ and *Q*(*v*, *T*_*e*_) accounting for NL current damping and electron heating have been calculated by the analytical integration of Rosebluth potentials and of ${\left(\partial_{t}f\right)}_{\text{coll}}$ in the weak coupling assumption. Note also that, for weak excitation *T*_*e*_ − *T*_0_ ≪ *T*_0_, |*v*/*v*_T0_|≪ 1, one recovers the RTA limit $F_{\gamma }≃1$ . Moreover, in such a limit *Q* ≃ − *γ*_th_(*T*_*e*_ − *T*_0_), and thus *γ*_th_ represents the hot electron relaxation rate to equilibrium via el–ph collisions.

## 3 NL response of Na thin films

**Figure 1:**
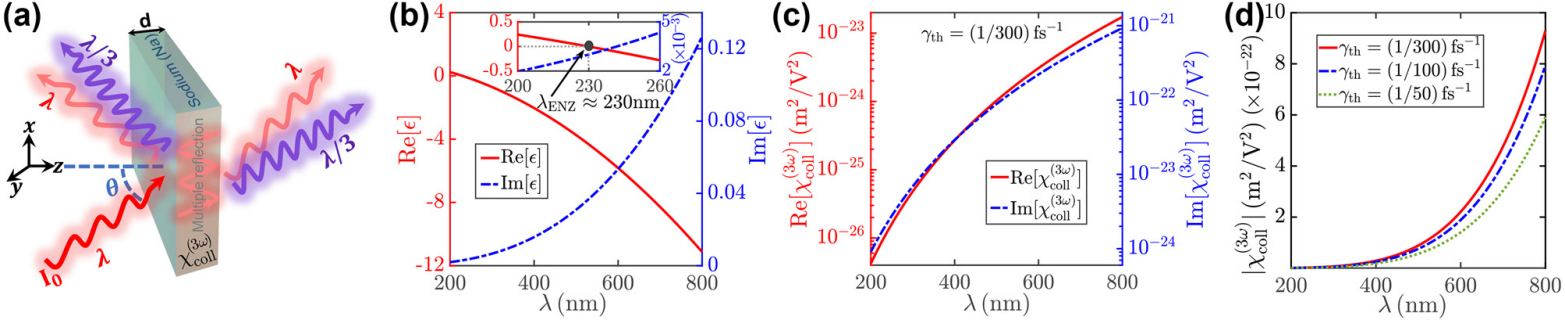
Na EM response. (a) Schematic of the considered Na-based thin film with thickness *d*, illuminated by a pump field with intensity *I*_0_ and vacuum wavelength *λ*, producing forward and backward THG at *λ*_THG_ = *λ*/3. (b) Dependence of real (red curve) and imaginary (dashed blue curve) parts of Na relative dielectric permittivity *ϵ*(*λ*). The ENZ wavelength of Na *λ*_ENZ_ where Re[*ϵ*(*λ*_ENZ_)] = 0 is indicated by the black solid circle in the figure inset. (c) Pump vacuum wavelength-dependence of the real (red curve) and imaginary (dashed blue curve) parts of the THG susceptibility $\chi_{\text{coll}}^{\left(3\omega \right)}\left(\lambda \right)$ . (d) Wavelength dependence of the THG NL susceptibility modulus $\left|\chi_{\text{coll}}^{\left(3\omega \right)}\left(\lambda \right)\right|$ for several distinct electron thermalization rates *γ*_th_ = 1/300, 1/100, 1/50 fs^−1^, indicated by full, dashed, and dotted curves, respectively.

We consider quasi-monochromatic pump ultrashort pulses with duration of the order of 500 fs, carrier angular frequency *ω* and associated electric field $E_{0}\left(r,t\right)=Re\left[A_{0}\left(t\right){\text{e}}^{\text{i}k_{0}\cdot r-\text{i}\omega t}\right]$ impinging over a Na-based thin film with thickness *d* immersed in air, see Figure 1a. We emphasize that, in view of the quasi-monochromatic regime of such ultrafast excitation, we adopt the slowly-varying envelope approximation (SVEA), where the pump vectorial envelope **A**_0_(*t*) is temporally modulated over a duration much longer than the single-cycle period 2*π*/*ω*. In turn, in the calculations below, the field envelopes are treated as time-independent quantities and the reported field intensities refer to peak intensities of the considered ultrashort pulses. Without any loss of generality, we assume that $k_{0}=k_{x}{\hat{e}}_{x}+\sqrt{\omega^{2}/c^{2}-k_{x}^{2}}{\hat{e}}_{z}$ lies in the *x* − *z* plane, where ${\hat{e}}_{x,y,z}$ indicate the *x*, *y*, *z* unit vectors, *k*_*x*_ = (*ω*/*c*)sin*θ* indicates the in-plane wave-vector component, and *θ* is the pump incidence angle, see Figure 1a. Owing to multiple reflections by the thin film interfaces, the pump electric field within the ENZ slab $E_{\text{in}}\left(r,t\right)=Re\left[A_{\text{in}}\left(r\right){\text{e}}^{-\text{i}\omega t}\right]$ is composed of both forward (FW) and backward (BW) waves, both incorporated within the vector field profile **A**_in_(**r**). Neglecting relativistic magnetic effects, we consider the weak EM excitation limit **v** ≃ **v**_1_ + **v**_3_, where |**A**_in_(**r**)| ≃ *o*(*ɛ*), |**v**_1_| ≃ *o*(*ɛ*), Δ*T* = *T*_*e*_ − *T*_0_ ≃ *o*(*ɛ* ^2^), |**v**_3_| ≃ *o*(*ɛ* ^3^), and *ɛ* is a small dummy parameter. Moreover, we focus on the local response where *n*(**r**, *t*) = *n*_0_, spatial derivatives in Eqs. (4a)–(4c) are neglected, and the electron temperature and mean velocity depend parametrically over the pump electric field at position **r**. In turn, developing a multiple scale expansion of HDEs, at first-order ≃*o*(*ɛ*) the electron mean velocity **v**_1_(**r**, *t*) remains unaffected by heating and satisfies the Drude model ∂_*t*_ **v**_1_ = −*γ* **v**_1_ − (*e*/*m*)**E**_in_, providing the solution $v_{1}\left(r,t\right)=Re\left[\left(-e/m\right){\left(\gamma -i\omega \right)}^{-1}A_{\text{in}}\left(r\right){\text{e}}^{-\text{i}\omega t}\right]$ . At second order ≃*o*(*ɛ* ^2^), the OOE electron temperature variation Δ*T*(**r**, *t*) is governed by $\partial_{t}\Delta T=\left(m/3k_{b}\right)\left(2\gamma -\gamma_{\text{th}}\right)v_{1}^{2}-\gamma_{\text{th}}\Delta T$ , whose stationary solution arising from the balance between heating (first term) and cooling (second term) provides

(6) $$\Delta T=Re\left[\frac{C_{H}A_{\text{in}}^{2}{\text{e}}^{-2\text{i}\omega t}}{{\left(\gamma -i\omega \right)}^{2}\left(\gamma_{\text{th}}-2i\omega \right)}\right]+\frac{C_{H}|A_{\text{in}}{|}^{2}}{\gamma_{\text{th}}\left(\gamma^{2}+\omega^{2}\right)},$$

where $C_{H}=e^{2}\left(2\gamma -\gamma_{\text{th}}\right)/6k_{b}m$ . Note that the OOE electron temperature oscillates with angular frequency 2*ω* around the intensity-dependent temperature shift produced in such a dynamical equilibrium between collision-induced heating upon illumination and relaxation via el–ph collisions. Finally, at third-order ≃*o*(*ɛ* ^3^), the electron mean velocity correction **v**_3_(**r**, *t*) is governed by

(7) $$\partial_{t}v_{3}=-\gamma v_{3}+\left(2\gamma -\gamma_{\text{th}}\right)\left[\frac{3\Delta T}{4T_{0}}+\frac{3mv_{1}^{2}}{20k_{b}T_{0}}\right]v_{1},$$

providing the solution $v_{3}=Re\left[s_{3}^{\left(3\omega \right)}A_{\text{in}}^{2}A_{\text{in}}{\text{e}}^{-3\text{i}\omega t}+\left(s_{3,+}^{\left(\omega \right)}|A_{\text{in}}{|}^{2}A_{\text{in}}+s_{3,-}^{\left(\omega \right)}A_{\text{in}}^{2}A_{\text{in}}^{*}\right){\text{e}}^{-\text{i}\omega t}\right]$ , where

(8a) $$s_{3}^{\left(3\omega \right)}=\frac{-e^{3}\left(2\gamma -\gamma_{\text{th}}\right)\left(5\gamma -\gamma_{\text{th}}-3i\omega \right)}{40m^{2}k_{b}T_{0}\left(\gamma_{\text{th}}-2i\omega \right)\left(\gamma -3i\omega \right){\left(\gamma -i\omega \right)}^{3}},$$

(8b) $$s_{3,+}^{\left(\omega \right)}=\frac{-e^{3}\left(2\gamma -\gamma_{\text{th}}\right)\left(5\gamma -\gamma_{\text{th}}\right)}{40m^{2}k_{b}T_{0}\gamma_{\text{th}}\left(\gamma^{2}+\omega^{2}\right){\left(\gamma -i\omega \right)}^{2}},$$

(8c) $$s_{3,-}^{\left(\omega \right)}=\frac{-e^{3}\left(2\gamma -\gamma_{\text{th}}\right)\left(5\gamma -\gamma_{\text{th}}-3i\omega \right)}{40m^{2}k_{b}T_{0}\left(\gamma_{\text{th}}-2i\omega \right)\left(\gamma^{2}+\omega^{2}\right){\left(\gamma -i\omega \right)}^{2}}.$$

Note that, owing to OOE electron dynamics, the electron mean velocity NL correction oscillates at *ω* (Kerr nonlinearity) and 3*ω* (THG). Kerr nonlinearity of the pump is highly amplified at ENZ conditions [17]. In our THG calculations below, we focus on pump excitation around 600 nm, where the real part of the relative dielectric permittivity of sodium is largely negative Re *ϵ* ≃ −10, see Figure 1b, and Kerr nonlinearity does not play a crucial role. In turn, in our theoretical framework we neglect Kerr nonlinearity of the pump, obtaining accurate results for *λ* > 330 nm (where Re *ϵ* < − 1, see Figure 1b), while at shorter pump wavelengths Kerr-induced spectral shifts of the pump absorbance produce NL modulations of THG efficiency that are not accounted in our theoretical framework. In such assumptions, the macroscopic polarization field **P**(**r**, *t*) can be calculated from the current density **J**(**r**, *t*) = −*en*_0_ **v**(**r**, *t*) = ∂_*t*_ **P**(**r**, *t*), providing $P\left(r,t\right)=\epsilon_{0}Re\left\{\left[\left(\epsilon -1\right){\text{e}}^{-\text{i}\omega t}+\chi_{\text{coll}}^{\left(3\omega \right)}A_{\text{in}}^{2}{\text{e}}^{-3\text{i}\omega t}\right]A_{\text{in}}\right\}$ , where $\epsilon \left(\omega \right)=1-\omega_{p}^{2}/\omega \left(\omega +i\gamma \right)$ is the Drude relative permittivity, $\omega_{p}=\sqrt{n_{0}e^{2}/\epsilon_{0}m}≃8.2\times 10^{15}$ rad/s is the sodium plasma frequency [27], *ϵ*_0_ is the dielectric permittivity of vacuum, and $\chi_{\text{coll}}^{\left(3\omega \right)}\left(\omega \right)=\left(m\omega_{p}^{2}/3ie\omega \right)s_{3}^{\left(3\omega \right)}\left(\omega \right)$ is the collision-induced THG NL susceptibility. Note that the ion mass *M* enters only in the relaxation rate *γ*_th_. In Figure 1b and c we depict the wavelength dependence of (b) real (red curve) and imaginary (dashed blue curve) parts of the relative dielectric permittivity of Na [*ϵ*(*λ*)] and (c) real (red curve) and imaginary (dashed blue curve) parts of its collision-induced THG NL susceptibility $\left[\chi_{\text{coll}}^{\left(3\omega \right)}\left(\lambda \right)\right]$ . Note that Na displays marked ENZ behavior at *λ*_ENZ_ = 2*πc*/*ω*_*p*_ ≃ 230 nm, where Im[*ϵ*(*λ*_ENZ_)] ≃ 10^−3^, see Figure 1b. In turn, conversely to ITO where ENZ behavior is attained in the IR and $|\epsilon \left(\lambda_{\text{ENZ}}^{\left(ITO\right)}\right)|≃0.5$ [17], for Na such a quantity becomes much smaller |*ϵ*(*λ*_ENZ_)| ≃ 10^−3^, showing great theoretical potential for ENZ functionality, see below. Note that the collision-induced THG NL susceptibility is highly dispersive, ranging from $|\chi_{\text{coll}}^{\left(3\omega \right)}|≃10^{-24}$ m^2^/V^2^ at *λ* = 200 nm to $|\chi_{\text{coll}}^{\left(3\omega \right)}|≃10^{-21}$ m^2^/V^2^ at *λ* = 800 nm. We emphasize that band anharmonicity and interband transitions (negligible for Na, for which linear optical response is well described by the Drude model) provide other contributions to the third-order NL susceptibility that we do not account here because the scope of the present work focuses on collision-induced NL dynamics. Furthermore, in our calculations we assume *γ*_th_ = (300 fs)^−1^, implying that hot electron relaxation in Na occurs over the same timescale of gold [35], [36]. More precise evaluation of *γ*_th_ requires pump-probe reflectivity measurements on Na by experimental schemes similar to the ones adopted for gold [35]. Because electron thermalization arises from electron-phonon scattering, it is highly dependent on sample pristinity. In turn, in practical experimental realizations, the precise electron thermalization rate will depend over the particular sample to be adopted. It is worth emphasizing that electron thermalization rate influences the third-order NL response of the system, see [Disp-formula j_nanoph-2023-0743_eq_008a] [Disp-formula j_nanoph-2023-0743_eq_008b] Eqs. (8), while it does not affect its linear response. As a consequence, the resonant THG mechanism theoretically described below in principle depends over the particular electron thermalization rate of the sample. However, we find that THG NL susceptibility is only weakly modulated by the electron thermalization rate, changing only by a factor 1.5 for (50 fs)^−1^ < *γ*_th_ < (300 fs)^−1^, see Figure 1d, where we depict the wavelength dependence of the THG NL susceptibility modulus $\left|\chi_{\text{coll}}^{\left(\lambda \right)}\right|$ for different electron thermalization rates *γ*_th_. Thus, the precise *γ*_th_ value does not lead to major quantitative THG NL susceptibility deviations.

## 4 THG in Na thin films

In order to account for both TM and TE pump excitation of the Na-based thin film, we set $A_{0}=A_{0}{\hat{n}}_{0}$ , where ${\hat{n}}_{0}=\alpha_{I}\cos\theta {\hat{e}}_{x}+\beta_{I}{\hat{e}}_{y}-\alpha_{I}\sin\theta {\hat{e}}_{z}$ is the impinging field unit vector, *A*_0_ is the electric field amplitude of the impinging wave, *β*_*I*_ = 1 − *α*_*I*_ and *α*_*I*_ = 0, 1 are dimension-less coefficients enabling selective TE (*α*_*I*_ = 0, *β*_*I*_ = 1) or TM (*α*_*I*_ = 1, *β*_*I*_ = 0) excitation. In turn, in the undepleted pump approximation, the pump field over all space is given by $E_{p}=Re\left[A_{p}\left(r\right){\text{e}}^{-\text{i}\omega t}\right]$ , where $A_{p}\left(r\right)=\left(A_{0}{\text{e}}^{\text{i}k_{0}\cdot r}+A_{r}{\text{e}}^{\text{i}k_{r}\cdot r}\right)\Theta \left(-z\right)+A_{\text{in}}\left(r\right)\Theta_{\text{in}}\left(z\right)+A_{t}{\text{e}}^{\text{i}k_{0}\cdot r}\Theta \left(z-d\right)$ , $A_{r}=A_{r}{\hat{n}}_{r}$ , ${\hat{n}}_{r}=\alpha_{I}\cos\theta {\hat{e}}_{x}+\beta_{I}{\hat{e}}_{y}+\alpha_{I}\sin\theta {\hat{e}}_{z}$ , *A*_*r*_ is the reflected field amplitude, $k_{r}=k_{x}{\hat{e}}_{x}-\sqrt{\omega^{2}/c^{2}-k_{x}^{2}}{\hat{e}}_{z}$ is the reflected wave-vector, $A_{\text{in}}=\sum_{s=\pm 1}A_{s}{\text{e}}^{\text{i}k_{s}\cdot r}$ is the electric field vector amplitude within the thin film, $k_{s}=k_{x}{\hat{e}}_{x}+s\sqrt{{\left(\omega /c\right)}^{2}\epsilon -k_{x}^{2}}{\hat{e}}_{z}$ are the FW (*s* = 1) and BW (*s* = −1) wave-vectors of the EM field with vector amplitudes $A_{s}=\alpha_{I}A_{s,x}{\hat{e}}_{x}+\beta_{I}A_{s,y}{\hat{e}}_{y}-s\alpha_{I}\left(\sin\theta /n_{\text{eff}}\right)A_{s,x}{\hat{e}}_{z}$ , $n_{\text{eff}}\left(\omega \right)=\sqrt{\epsilon -\sin^{2}\theta }$ , *A*_*s*,{*x*,*y*}_ are the transverse field components in Na, $A_{t}=A_{t}{\hat{n}}_{0}$ , *A*_*t*_ is the transmitted field amplitude, Θ_in_(*z*) = Θ(*z*) − Θ(*z* − *d*), and Θ(*z*) is the Heaviside step function. The pump induction magnetic field over all space is calculated from the Faraday law in local form, providing $B_{p}=Re\left[\left(1/i\omega \right)\nabla \times A_{p}\left(r\right){\text{e}}^{-\text{i}\omega t}\right]$ . Thus, the unknown transmitted, reflected, FW and BW pump field amplitudes are obtained from boundary conditions (BCs) for the continuity of the magnetic field, the tangential electric field components, and the normal component of the displacement vector at the interfaces *z* = 0, *d*. Such BCs provide two uncoupled 4 × 4 inhomogeneous systems of equations for TM and TE amplitudes, explicitly given by

(9) $$\alpha_{I}{\hat{M}}_{\text{TM}}^{\left(\omega ,\theta \right)}V_{\text{TM}}+\beta_{I}{\hat{M}}_{\text{TE}}^{\left(\omega ,\theta \right)}V_{\text{TE}}=\alpha_{I}W_{\text{TM}}+\beta_{I}W_{\text{TE}},$$

where $V_{\text{TM}}={\left[A_{r}\;A_{+,x}\;A_{-,x}\;A_{t}\right]}^{T}$ , **W**_TM_ = −*A*_0_[cos*θ* 1 0 0]^T^, $V_{\text{TE}}={\left[A_{r}\;A_{+,y}\;A_{-,y}\;A_{t}\right]}^{T}$ , **W**_TE_ = *A*_0_[−1 cos*θ* 0 0]^T^, $\eta_{\pm }\left(\omega ,\theta \right)={\text{e}}^{\pm \text{i}\omega n_{\text{eff}}d/c}$ , *η*_0_(*ω*, *θ*) = e^i*ω*cos*θd*/*c*^ and

(10a) $${\hat{M}}_{\text{TM}}^{\left(\omega ,\theta \right)}=\left[\begin{array}{cccc} \cos\theta & \;-1 & \;-1 & 0\\ -1 & \;-\frac{\epsilon }{n_{\text{eff}}} & \frac{\epsilon }{n_{\text{eff}}} & 0\\ 0 & \eta_{+} & \eta_{-} & \;-\eta_{0}\cos\theta \\ 0 & \;-\frac{\epsilon }{n_{\text{eff}}}\eta_{+} & \frac{\epsilon }{n_{\text{eff}}}\eta_{-} & \eta_{0} \end{array}\right],$$

(10b) $${\hat{M}}_{\text{TE}}^{\left(\omega ,\theta \right)}=\left[\begin{array}{cccc} 1 & \;-1 & \;-1 & 0\\ \cos\theta & n_{\text{eff}} & \;-n_{\text{eff}} & 0\\ 0 & \eta_{+} & \eta_{-} & \;-\eta_{0}\\ 0 & \;-n_{\text{eff}}\eta_{+} & n_{\text{eff}}\eta_{-} & \eta_{0}\cos\theta \end{array}\right].$$

**Figure 2:**
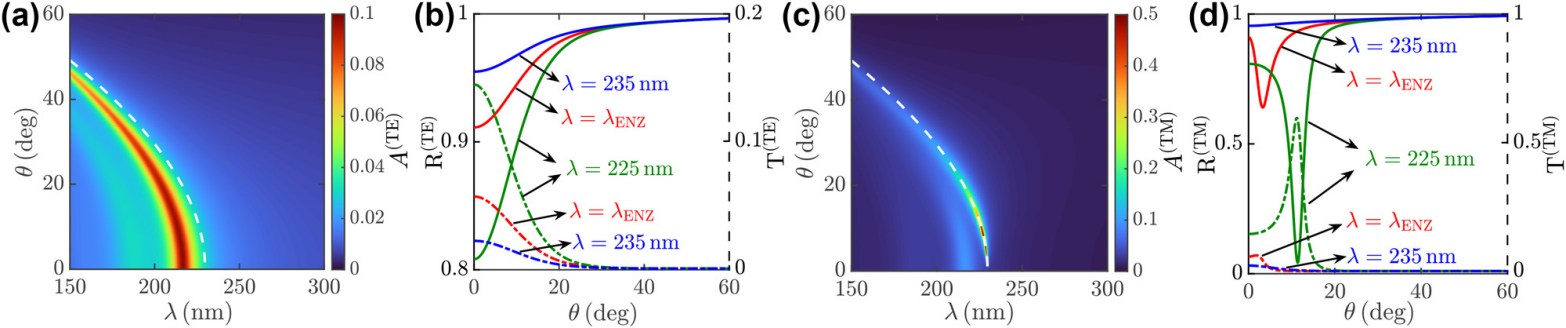
Linear response of Na-based thin films. Dependence over the pump wavelength *λ* and incidence angle *θ* of the (a, c) absorbance *A* ^(TE,TM)^ and (b, d) reflectance *R* ^(TE,TM)^ (full curves) and transmittance *T* ^(TE,TM)^ (dahsed curves) for (a, b) TE and (c, d) TM excitation of a Na-based thin film with thickness *d* = 300 nm. The dashed white curves in (a, c) indicate the NZI dispersion curve *θ*_NZI_(*λ*).

We emphasize that, for the pump field, all the elements of the TM ${\hat{M}}_{\text{TM}}^{\left(\omega ,\theta \right)}$ and TE ${\hat{M}}_{\text{TE}}^{\left(\omega ,\theta \right)}$ scattering matrices are evaluated at the pump angular frequency *ω* and incident angle *θ*, indicated in their superscripts. Also, note that the formalism above refers to single TE (*α*_*I*_ = 0) or TM (*α*_*I*_ = 1) excitation (*β*_*I*_ = 1 − *α*_*I*_ in both cases), but is not suited to treat mixed polarization states, requiring a more general treatment accounting for the polarization dynamics of reflected and transmitted waves. Inverting the system above, one gets all the pump field amplitudes for TE and TM excitation. Figure 2 illustrates the dependence of the pump (a, c) absorbance *A* = 1 − *R* − *T* and (b, d) reflectance *R* = 1 − |*A*_*r*_|^2^/|*A*_0_|^2^ and transmittance *T* = 1 − |*A*_*t*_|^2^/|*A*_0_|^2^ over the pump wavelength *λ* and incidence angle *θ* for (a, b) TE (*α*_*I*_ = 0, *β*_*I*_ = 1) and (c, d) TM (*α*_*I*_ = 1, *β*_*I*_ = 0) excitation of a Na-based thin film of thickness *d* = 300 nm. Note that absorbance, reflectance, and transmittance heavily depend over both the pump wavelength and incidence angle when *λ* < *λ*_ENZ_ owing to NZI resonance occurring when 0 < Re[*ϵ*(*λ*)] < 1 and $Re\left[n_{\text{eff}}^{2}\right]=0$ , which provides the dispersion relation $\theta_{\text{NZI}}\left(\lambda \right)=asin\sqrt{Re\left[\epsilon \left(\lambda \right)\right]}$ , indicated by the white dashed curves in Figure 2a and c. Note that the maximum absorbance is attained for TM excitation exactly at the NZI resonance, see Figure 2c. Moreover, due to the “metal-dielectric” transition occurring at *λ*_ENZ_, for *λ* < *λ*_ENZ_ Fabry–Perot absorption resonances are excited by both TE and TM impinging polarization, see Figure 2a and c, for *θ* < *θ*_NZI_(*λ*). The pump field THG NL polarization within the Na-based thin film is given by $P_{\text{nl}}\left(r,t\right)=Re\left[p_{\text{nl}}\left(z\right){\text{e}}^{3\text{i}k_{x}x-3\text{i}\omega t}\right]$ , where

(11) $$\begin{array}{ll} p_{\text{nl}}\left(z\right) & =\epsilon_{0}\chi_{\text{coll}}^{\left(3\omega \right)}\underset{s=\pm 1}{\sum }\left[A_{s}^{2}A_{s}{\text{e}}^{3\text{i}s\phi_{\omega }\left(z\right)}\right.\\ & \;\left.+\left[A_{s}^{2}A_{-s}+2\left(A_{s}\cdot A_{-s}\right)A_{s}\right]{\text{e}}^{\text{i}s\phi_{\omega }\left(z\right)}\right], \end{array}$$

and *ϕ*_*ω*_(*z*) = *ωn*_eff_(*ω*)*z*/*c*. Such NL polarization generates 3*ω* radiation due to the associated polarization (i) current density **J**_nl_ = ∂_*t*_ **P**_nl_, and (ii) volume *ρ*_nl_ = −∇⋅**P**_nl_ and (iii) surface $\sigma_{\text{nl},\pm }=\pm P_{\text{nl}}\left(z=d\left[1\pm 1\right]/2\right)\cdot {\hat{e}}_{z}$ charge densities appearing as inhomogeneous terms for the THG signal in (i, ii) Maxwell’s equations within the thin film and (iii) BCs at the two interfaces *z* = 0, *d*. We take the Ansatz $E_{\text{THG}}\left(r,t\right)=Re\left[e_{\text{THG}}\left(z\right){\text{e}}^{3\text{i}k_{x}x-3\text{i}\omega t}\right]$ , where $e_{\text{THG}}=\left[a_{r}{\text{e}}^{3\text{i}k_{r,z}z}{\hat{n}}_{r}\Theta \left(-z\right)+A_{\text{THG}}^{\text{in}}\left(z\right)\Theta_{\text{in}}\left(z\right)+a_{t}{\text{e}}^{3\text{i}k_{0,z}z}{\hat{n}}_{0}\Theta \left(z\right)\right]$ . Hence, the THG induction magnetic field is obtained from the Faraday law in local form, providing $B_{\text{THG}}\left(r,t\right)=Re\left\{\left(1/3i\omega \right)\nabla \times \left[e_{\text{THG}}\left(z\right){\text{e}}^{3\text{i}k_{x}x}\right]{\text{e}}^{-3\text{i}\omega t}\right\}$ . Inserting this Ansatz in Maxwell’s equations accounting for **J**_nl_ and *ρ*_nl_, we obtain an inhomogeneous ordinary differential equation for the THG field within the Na-based thin film, explicitly given by

(12) $$\begin{array}{ll} & \frac{d^{2}}{dz^{2}}A_{\text{THG}}^{\text{in}}+9\frac{\omega^{2}}{c^{2}}n_{\text{eff}}^{2}\left(3\omega \right)A_{\text{THG}}^{\text{in}}\\ & \;=\underset{s=\pm 1}{\sum }\left[\Lambda_{s}{\text{e}}^{\text{i}s\phi_{\omega }\left(z\right)}+\Omega_{s}{\text{e}}^{3\text{i}s\phi_{\omega }\left(z\right)}\right], \end{array}$$

where

$$\Lambda_{s}=\frac{\omega^{2}}{c^{2}}\chi_{\text{coll}}^{\left(3\omega \right)}\left(\omega \right)\underset{s=\pm 1}{\sum }\left\{\frac{4\sin\theta }{\epsilon \left(3\omega \right)}{ϒ}_{s,x}{\hat{n}}_{\text{nl}}-9\Gamma_{s}\right\},$$

$\Omega_{s}\left(\omega ,\theta \right)=-9\omega^{2}\chi_{\text{coll}}^{\left(3\omega \right)}A_{s}^{2}A_{s}/c^{2}$ , ${\hat{n}}_{\text{nl}}=\left[3\sin\theta {\hat{e}}_{x}+sn_{\text{eff}}\left(\omega \right){\hat{e}}_{z}\right]$ , $\Gamma_{s}=\left[A_{s}^{2}A_{-s}+2\left(A_{s}\cdot A_{-s}\right)A_{s}\right]$ , and **ϒ**_*s*_ = **Γ**_*s*_ − (**A**_*s*_ ⋅**A**_−*s*_)**A**_*s*_. Thus, we obtain the solution $A_{\text{THG}}^{\text{in}}=\underset{s=\pm 1}{\sum }\left[{\tilde{\Lambda }}_{s}{\text{e}}^{\text{i}s\phi_{\omega }}+{\tilde{\Omega }}_{s}{\text{e}}^{3\text{i}s\phi_{\omega }}+a_{s}{\text{e}}^{3\text{i}s\phi_{3\omega }}\right]$ , where ${\tilde{\Lambda }}_{s}=\Lambda_{s}c^{2}/\omega^{2}\left[9n_{\text{eff}}^{2}\left(3\omega \right)-n_{\text{eff}}^{2}\left(\omega \right)\right]$ and ${\tilde{\Omega }}_{s}=\Omega_{s}c^{2}/9\omega^{2}\left[n_{\text{eff}}^{2}\left(3\omega \right)-n_{\text{eff}}^{2}\left(\omega \right)\right]$ , $a_{s}=\alpha_{\text{I}}a_{s,x}{\hat{e}}_{x}+\beta_{\text{I}}a_{s,y}{\hat{e}}_{y}-s\alpha_{\text{I}}\left[\sin\theta /n_{\text{eff}}\left(3\omega \right)\right]a_{s,x}{\hat{e}}_{z}$ , and *a*_*s*,{*x*,*y*}_ are homogeneous amplitudes yet to be determined.

In order to obtain the unknown THG field amplitudes, we impose BCs at 3*ω* (accounting also for the polarization surface charge densities produced by **p**_nl_), explicitly given by

(13a) $$\nabla \times \left[e_{\text{THG}}\left(z_{0}^{+}\right){\text{e}}^{3\text{i}k_{x}x}\right]=\nabla \times \left[e_{\text{THG}}\left(z_{0}^{-}\right){\text{e}}^{3\text{i}k_{x}x}\right],$$

(13b) $${\hat{e}}_{z}\times \left[e_{\text{THG}}\left(z_{0}^{+}\right){\text{e}}^{3\text{i}k_{x}x}-e_{\text{THG}}\left(z_{0}^{-}\right){\text{e}}^{3\text{i}k_{x}x}\right]=0,$$

(13c) $${\hat{e}}_{z}\cdot \left[d_{\text{THG}}\left(z_{0}^{+}\right)-d_{\text{THG}}\left(z_{0}^{-}\right)\right]=p_{z_{0}}p_{\text{nl}}\left(z_{0}\right)\cdot {\hat{e}}_{z},$$

where $p_{z_{0}}=\left(2z_{0}-d\right)/d$ , *z*_0_ = 0, *d* and **d**_THG_ = *ϵ*_0_{1 + [*ϵ*(3*ω*) − 1]Θ_in_(*z*)}**e**_THG_. Such BCs provide two uncoupled 4 × 4 inhomogeneous systems of equations for TM and TE amplitudes of the THG signal, explicitly given by

(14) $$\alpha_{I}{\hat{M}}_{\text{TM}}^{\left(3\omega ,\theta \right)}{\tilde{V}}_{\text{TM}}+\beta_{I}{\hat{M}}_{\text{TE}}^{\left(3\omega ,\theta \right)}{\tilde{V}}_{\text{TE}}=\alpha_{I}{\tilde{W}}_{\text{TM}}+\beta_{I}{\tilde{W}}_{\text{TE}},$$

where

$${\tilde{V}}_{\text{TM}}=\left[\begin{array}{c} a_{r}\\ a_{+,x}\\ a_{-,x}\\ a_{t} \end{array}\right],\;{\tilde{V}}_{\text{TE}}=\left[\begin{array}{c} a_{r}\\ a_{+,y}\\ a_{-,y}\\ a_{t} \end{array}\right],\;{\tilde{W}}_{TK}=\left[\begin{array}{c} {\tilde{W}}_{TK}^{\left(1\right)}\\ {\tilde{W}}_{TK}^{\left(2\right)}\\ {\tilde{W}}_{TK}^{\left(3\right)}\\ {\tilde{W}}_{TK}^{\left(4\right)} \end{array}\right],$$

*K* = *E*, *M* is an index labelling TE/TM excitation, ${\tilde{W}}_{\text{TE}}={\left[{\tilde{W}}_{y}^{\left(1\right)}\;-{\tilde{W}}_{y}^{\left(2\right)}\;{\tilde{W}}_{y}^{\left(3\right)}\;{\tilde{W}}_{y}^{\left(4\right)}\right]}^{T}$ , ${\tilde{W}}_{\text{TM}}={\left[{\tilde{W}}_{x}^{\left(1\right)}\;\left({\tilde{W}}_{x}^{\left(2\right)}-\tilde{\sin}\theta {\tilde{W}}_{z}^{\left(1\right)}\right)\;{\tilde{W}}_{x}^{\left(3\right)}\;\left({\tilde{W}}_{x}^{\left(4\right)}+\sin\theta {\tilde{W}}_{z}^{\left(3\right)}\right)\right]}^{T}$ , ${\tilde{W}}^{\left(1\right)}=\sum_{s=\pm 1}\left[{\tilde{\Omega }}_{s}+{\tilde{\Lambda }}_{s}\right]$ , ${\tilde{W}}^{\left(2\right)}=n_{\text{eff}}\left(\omega \right)\sum_{s=\pm 1}s\left[{\tilde{\Omega }}_{s}+\left(1/3\right){\tilde{\Lambda }}_{s}\right]$ , ${\tilde{W}}^{\left(3\right)}=-\sum_{s=\pm 1}\left[{\tilde{\Omega }}_{s}{\text{e}}^{3\text{i}s\phi_{\omega }\left(d\right)}+{\tilde{\Lambda }}_{s}{\text{e}}^{\text{i}s\phi_{\omega }\left(d\right)}\right]$ , and ${\tilde{W}}^{\left(4\right)}=n_{\text{eff}}\left(\omega \right)\sum_{s=\pm 1}s\left[{\tilde{\Omega }}_{s}{\text{e}}^{3\text{i}s\phi_{\omega }\left(d\right)}+\left(1/3\right){\tilde{\Lambda }}_{s}{\text{e}}^{\text{i}s\phi_{\omega }\left(d\right)}\right]$ .

**Figure 3:**
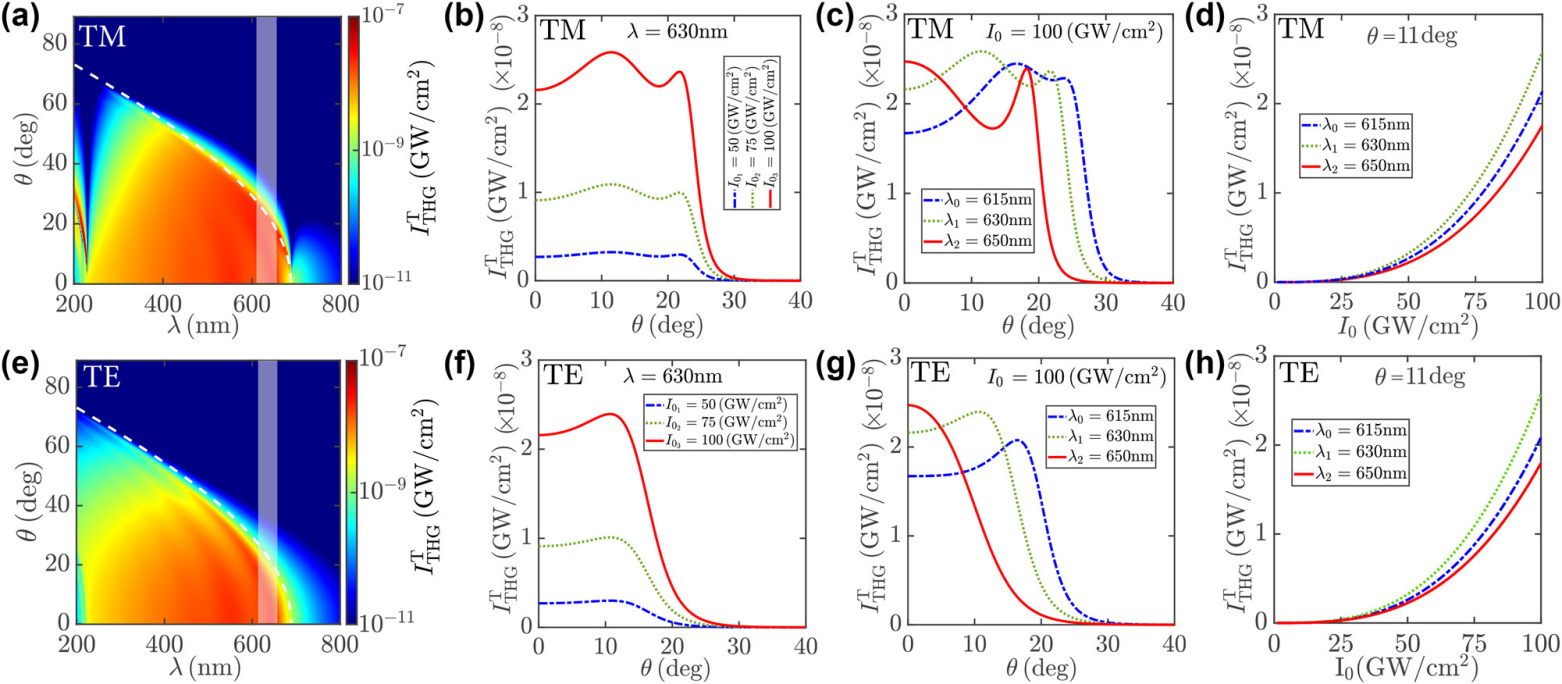
THG by Na-based thin films. (a, e) Dependence of the FW intensity of the THG signal $I_{\text{THG}}^{T}$ over the pump wavelength *λ* and incidence angle *θ* for fixed pump intensity *I*_0_ = 100 GW/cm^2^. (b, c, f, g) Dependence of $I_{\text{THG}}^{T}$ over the pump incidence angle *θ* for (b, f) fixed pump wavelength *λ* = 630 nm and several intensities *I*_0_ = 50, 75, 100 GW/cm^2^, and (c, g) fixed pump intensity *I*_0_ = 100 GW/cm^2^ and several wavelengths *λ* = 615, 630, 650 nm [highlighted by the gray shaded area in (a, e)]. (d, h) Dependence of $I_{\text{THG}}^{T}$ over the pump intensity *I*_0_ for fixed incidence angle *θ* = 11° and several wavelengths *λ* = 615, 630, 650 nm [highlighted by the gray shaded area in (a, e)]. All plots refer to either (a–d) TM or (e–h) TE polarization of the pump field for fixed thin film thickness *d* = 300 nm.

**Figure 4:**
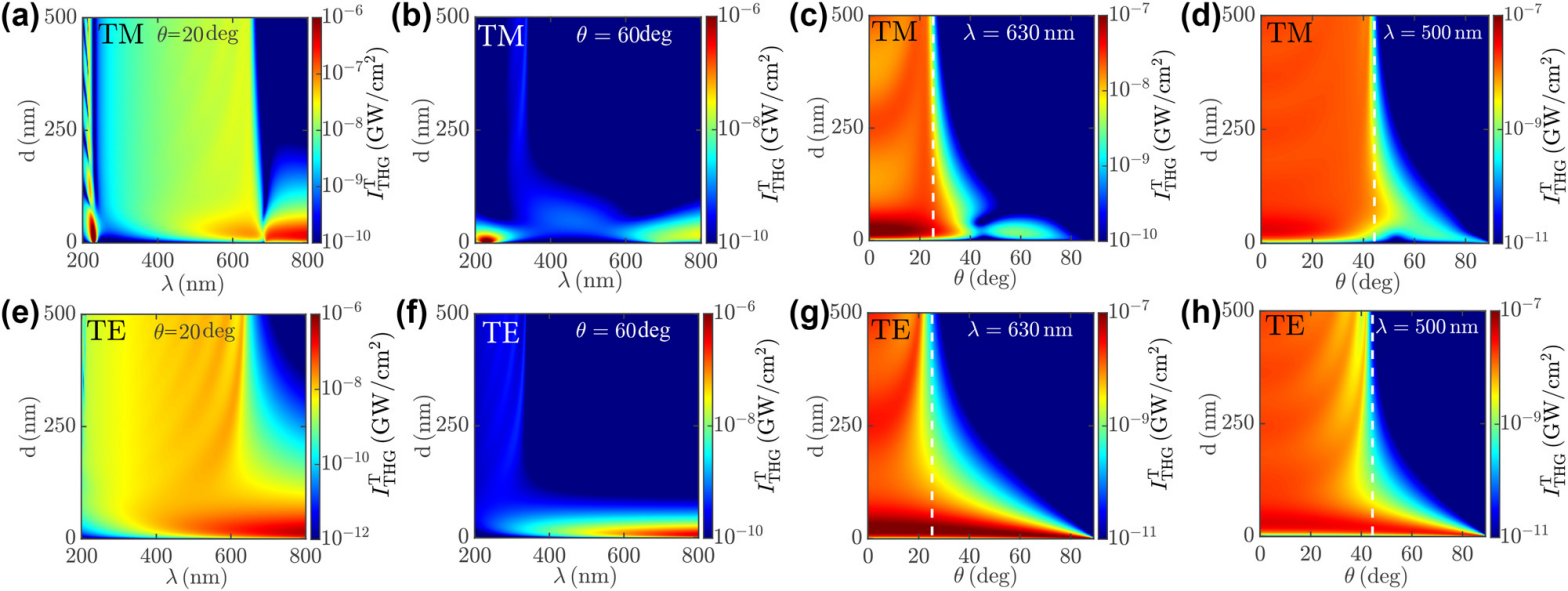
THG manipulation by Na-based thin films. (a, b, e, f) Dependence of the FW intensity of the THG signal $I_{\text{THG}}^{T}$ over the thin film thickness *d* and the pump wavelength *λ* for incidence angle (a, e) *θ* = 20° and (b, f) *θ* = 60°. (c, d, g, h) Dependence of $I_{\text{THG}}^{T}$ over the thin film thickness *d* and the pump incidence angle *θ* for wavelength (c, g) *λ* = 630 nm and (d, h) *λ* = 500 nm. All plots refer to either (a–d) TM or (e–h) TE polarization of the pump field. The white dashed vertical lines in (c, d, g, h) indicate the NZI critical angle *θ*_NZI_(*λ*/3). All plots are obtained for fixed pump intensity *I*_0_ = 100 GW/cm^2^.

**Figure 5:**
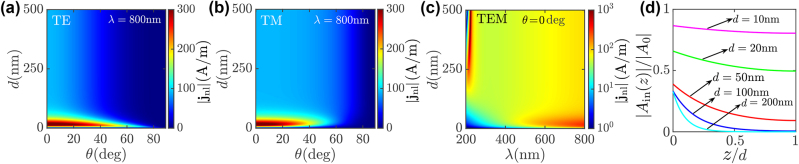
Surface-like NL interaction. (a–c) Dependence of the effective NL current per unit length modulus |**j**_nl_| over the thin film thickness *d* and the pump incidence angle *θ* and wavelength *λ*. (a, b) Dependence of |**j**_nl_| over *θ* and *d* for fixed pump wavelength *λ* = 800 nm and (a) TE and (b) TM polarization. (c) Dependence of |**j**_nl_| over *λ* and *d* at normal incidence. (d) Spatial dependence of the pump electric field profile modulus within the Na-based thin film rescaled to the impinging amplitude |**A**_in_(*z*)|/|*A*_0_| at transverse electromagnetic (TEM) normal incidence for fixed wavelength *λ* = 800 nm and several thicknesses *d* = 10, 20, 50, 100, 200 nm. All plots are obtained for fixed pump intensity *I*_0_ = 100 GW/cm^2^.

Note that, analogously to the pump, see Eq. (9), the THG field amplitudes satisfy a similar inhomogeneous system, see Eq. (14). However, we emphasize that in Eq. (14) all scattering matrices [ ${\hat{M}}_{\text{TM,TE}}^{\left(3\omega ,\theta \right)}$ ] elements are calculated at angular frequency 3*ω*, as labelled in the superscripts, and account for the THG radiation produced by **P**_nl_ within the film. Inverting Eq. (14) we obtain all the THG field amplitudes for either TE or TM polarization of the impinging pump with intensity *I*_0_ = (1/2)*ϵ*_0_ *c*|*A*_0_|^2^. In Figure 3 we illustrate the dependence of the FW intensity of the THG signal $I_{\text{THG}}^{T}=\left(1/2\right)\epsilon_{0}c|a_{t}{|}^{2}$ over the pump wavelength *λ*, incidence angle *θ* and intensity *I*_0_. We observe that, in spite of the reduced THG NL susceptibility $\chi_{\text{coll}}^{\left(3\omega \right)}$ by ≃3 orders of magnitude at *λ* ≃ 200 nm with respect to *λ* ≃ 800 nm, see Figure 1c, the THG process is amplified by ≃10^2^ at *λ* ≃ 200 nm owing to ENZ field enhancement of the pump for TM polarization, see Figure 3a, implying ≃10^5^ THG amplification by the NZI resonance of a Na-based thin film of thickness *d* = 300 nm. Because the FW THG intensity $I_{\text{THG}}^{T}\propto I_{0}^{2}$ scales with the square of the impinging pump intensity, see Figure 3d and h, this implies an effective averaged field enhancement >10 by a thin film of thickness *d* = 300 nm at *λ*_ENZ_. Moreover, for TM polarization we observe THG amplification also for *λ* < 3*λ*_ENZ_ due to NZI resonance of the THG signal field itself, see the dashed white curve in Figure 3a indicating the NZI dispersion relation *θ*_NZI_(*λ*/3). We emphasize that such signal amplification does not arise from the non-resonant pump field enhancement, but rather from the boost of THG radiation by microscopic dipoles immersed in an ENZ environment. This is mathematically accounted by the Gauss law in local form for the THG signal ∇ ⋅ **e**_THG_ = −∇ ⋅ **p**_nl_/*ϵ*_0_ *ϵ*(3*ω*), accounting for the boost of THG polarization volume charge density by ≃3 orders of magnitude at the ENZ resonance, where |*ϵ*(3*ω*)| ≃ 10^−3^, see Figure 1b. In addition to NZI resonance, several Fabry–Perot resonances of the THG signal for *λ* < 3*λ*_ENZ_ produce broadband THG amplification ×50 for both TE and TM polarization of the impinging pump, see Figure 3b, c, f, and g. Such a broadband functionality is enabled by the “metal–dielectric” transition [23], [24] of the ENZ material (for THG wavelength *λ*/3 < *λ*_ENZ_) that, thanks to the reduced absorption of Na, can support Fabry–Perot resonances with quality factor almost comparable to transparent photonic materials. Indeed, we observe a strong dependence of the THG conversion efficiency over the Na-based film thickness *d*. We investigate systematically the FW THG intensity $I_{\text{THG}}^{T}$ produced by the thin film, see Figure 4, where we illustrate its dependence over the thin film thickness *d*, the pump wavelength *λ* and the incidence angle *θ* for both TM and TE polarization of the pump field. Note that enhanced THG at the NZI dispersion relation *θ*_NZI_(*λ*/3) is heavily modulated by the thin film thickness *d* for TM polarization of the pump, see Figure 4a and b. Indeed, at *λ* ≃ 200 nm for every fixed incidence angle *θ*, we observe an optimal film thickness $\bar{d}≃20-50$ nm that decreases when the incidence angle increases. This is due to destroying interference of THG by FW and BW waves, which gets minimised when *d* is reduced. Moreover, for both TM, see Figure 4a–d, and TE, see Figure 4e–h, pump polarization and *λ* < 3*λ*_ENZ_, we observe that Fabry–Perot resonances at *λ*/3 produce optimal THG conversion efficiency at several thicknesses ${\bar{d}}_{n}$ that depend over the incidence angle *θ* owing to the modified optical path of THG waves. Moreover, we observe optimal THG amplification also for *θ* > *θ*_NZI_(*λ*/3) where the Na-based thin film optically behaves as a metal, see the white dashed vertical lines in Figure 4c and g indicating the NZI critical angle *θ*_NZI_(*λ*/3). Such behavior is not produced by THG resonance, but rather by maximisation of the pump field intensity within the thin film. Indeed, when $\lambda >\lambda_{\text{NZI}}=\theta_{\text{NZI}}^{-1}\left(\theta \right)$ , see Figures 2 and 3a and e, the Na-based thin film is highly reflective for the pump field. In turn, in such conditions, reducing the film thickness does not reduce the pump intensity within Na, but rather amplifies it due to back-reflection from the *z* = *d* interface. In order to account for this effect, we calculate the averaged NL current density per unit length $J_{\text{nl}}^{\left(\Lambda \right)}\left(x,t\right)=Re\left[j_{\text{nl}}{\text{e}}^{3\text{i}k_{x}x-3\text{i}\omega t}\right]=\int_{0}^{d}Re\left[-3i\omega p_{\text{nl}}\left(z\right){\text{e}}^{3\text{i}k_{x}x-3\text{i}\omega t}\right]dz$ , where **j**_nl_ is the effective vectorial amplitude of $J_{\text{nl}}^{\left(\Lambda \right)}$ . In Figure 5a–c, we depict the dependence of |**j**_nl_| over the film thickness *d* and the pump incidence angle *θ* and wavelength *λ*. Note that the *d* dependence of THG amplification for *λ* ≃ 800 nm, see Figure 4a, b, e, and f, coincides with the |**j**_nl_| enhancement dependence observed in Figure 5a–c. In turn, THG amplification for *λ* > 3*λ*_ENZ_ at small film thicknesses arises from the enhancement of the effective NL current density per unit length $J_{\text{nl}}^{\left(\Lambda \right)}$ . The physical origin of such unusual behavior stems from the high reflectivity of Na in such spectral range, producing evanescent pump field within the slab, see Figure 5d, where we illustrate the spatial dependence of the pump field within the film. Note that, when the thickness *d* decreases, the pump field within the film increases due to enhanced back-reflection of evanescent waves and in turn |**j**_nl_| is increased up to a critical thickness $\bar{d}≃40$ nm, where the field becomes uniform and |**j**_nl_| ≃ |**A**_in_|*d* starts decreasing due to the reduced film thickness, vanishing for *d* = 0. Overall, for both TE and TM pump polarization, we observe two distinct incidence angles where resonant THG amplifies the FW signal by ≃10^4^, and a similar behavior is observed also for the BW THG signal.

**Figure 6:**
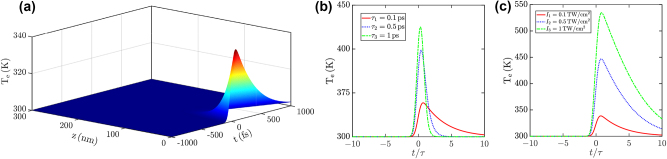
Thermal dynamics in Na-based thin films. (a) Dependence of the electron temperature *T*_*e*_ over the longitudinal position within the thin film *z* and the excitation time *t* for fixed pump peak intensity *I*_0_ = 100 GW/cm^2^ and pulse duration *τ* = 0.1 ps. (b) Temporal evolution of *T*_*e*_ for fixed pump peak intensity *I*_0_ = 100 GW/cm^2^, longitudinal position *z* = 0 and several pulse durations *τ* = 0.1, 0.5, 1 ps. (c) Temporal evolution of *T*_*e*_ for several pump peaks intensities *I*_0_ = 0.1, 0.5, 1 TW/cm^2^ and fixed pulse duration *τ* = 0.1 ps and longitudinal position *z* = 0. All plots are obtained at TEM normal incidence and for fixed pump carrier wavelength *λ* = 690 nm.

Such a surface-like NL interaction arises from thermal nonlinearity, producing THG by 2*ω* oscillations of the OOE electron temperature. In order to illustrate the ultrafast response of such an NL process, we consider an impinging pump optical envelope $A_{0}\left(t\right)=\sqrt{2I_{0}/\epsilon_{0}c}e^{-2\ln2t^{2}/\tau^{2}}{\hat{e}}_{x}$ with peak intensity *I*_0_ and temporal full width at half maximum (FWHM) *τ* at the *z* = 0 position producing within the Na-based thin film both FW and BW waves accounted by the solutions of Eq. (9) in the SVEA. We solve numerically Eq. (4) under such a driving field at every *z* position by an adaptive step fourth-order Runge–Kutta algorithm, enabling us to calculate the spatio-temporal dependence of the OOE electron temperature, illustrated in Figure 6a for *I*_0_ = 100 GW/cm^2^, *τ* = 0.1 ps and carrier wavelength *λ* = 690 nm. Note that, in such excitation conditions, the OOE electron temperature increases only within the initial propagation distance ≃50 nm, confirming the surface-like NL interaction. In Figure 6b and c we illustrate the temporal evolution of the OOE electron temperature *T*_*e*_ for (b) several distinct pulse duration FWHM *τ* and fixed peak intensity *I*_0_ = 100 GW/cm^2^, and (c) several distinct peak intensities and fixed pulse duration FWHM *τ* = 0.1 ps. We observe that the ultrafast electron heating produces OOE electron temperature increase of only ≃40 K owing to the slow damping rate *γ* ≃ 24.6 ps^−1^, producing reduced EM radiation absorption and heating.

## 5 Conclusions

In summary, collision-induced third-order nonlinearity produces efficient THG in Na-based thin films thanks to highly dispersive NL THG susceptibility $\chi_{\text{coll}}^{\left(3\omega \right)}$ ranging from $|\chi_{\text{coll}}^{\left(3\omega \right)}|≃10^{-24}$ m^2^/V^2^ at *λ* ≃ 200 nm to $|\chi_{\text{coll}}^{\left(3\omega \right)}|≃10^{-21}$ m^2^/V^2^ at *λ* ≃ 800 nm, where *λ* is the optical pump angular frequency. Moreover, Na displays great theoretical potential for highly efficient NZI features at *λ*/3 < *λ*_ENZ_ ≃ 230 nm thanks to mitigated absorption producing Im[*ϵ*(*λ*_ENZ_)] ≃ 10^−3^, about 500 times smaller than ITO. We find that, thanks to such extreme NZI behavior and the excitation of diverse Fabry–Perot THG resonances, it is possible to attain resonant THG over a broad spectral range by tuning the pump incidence angle *θ* for *λ* < 3*λ*_ENZ_. NZI resonances produce a giant THG enhancement by ≃10^4^ at peculiar thicknesses $\bar{d}≃40$ nm dictated by optimal pump field penetration within the highly reflective Na-based thin film. For pump intensities *I*_0_ ≃ 100 GW/cm^2^ at *λ* ≃ 800 nm, we observe FW THG intensities $I_{\text{THG}}^{T}≃1$ kW/cm^2^. Our results indicate that Na-based thin films are promising for the development of integrated UV sources and innovative spectroscopy schemes at the nanoscale.
